# The combination of osimertinib and chemotherapy successfully enabled surgical resection of locally advanced lung adenocarcinoma: a Case Report

**DOI:** 10.3389/fphar.2026.1695807

**Published:** 2026-05-12

**Authors:** Cheng-Yi Lin, Qun-Xian Zhang, Dan Li, Tao Zeng, Jun Zhang, Qiang Guo, Xiang-Yu Luo, Tao Liu

**Affiliations:** 1 Department of Cardiothoracic Surgery, Taihe Hospital, Hubei University of Medicine, Shiyan, Hubei, China; 2 Department of Oncology, Taihe Hospital, Hubei University of Medicine, Shiyan, Hubei, China

**Keywords:** CT, EGFR, lobectomy, lung adenocarcinoma, Osimertinib

## Abstract

We present the case of a 33-year-old female with a 4.3 × 3.2 cm high-density mass in the apical segment of the right upper lobe, detected on non-contrast chest CT, which showed suspected invasion of the chest wall and other tissues. CT-guided lung biopsy confirmed lung adenocarcinoma, and genetic testing revealed an EGFR exon 19 deletion. The patient received neoadjuvant therapy with oral Osimertinib combined with chemotherapy. After 4 months of treatment, she underwent thoracoscopic right upper lobectomy, mediastinal lymph node dissection, and pleural adhesion lysis. Postoperative management included anti-infection therapy, expectorants, nebulization, and analgesia. The patient recovered well and was discharged on postoperative day five, with good general condition at follow-up. This case illustrates that neoadjuvant targeted therapy combined with chemotherapy can convert locally unresectable lung cancer to a resectable status and improve operative feasibility, thus offering valuable insights for clinical management.

## Background

Lung adenocarcinoma represents a major histological subtype of non-small cell lung cancer (Wu et al., 2024; Dong et al., 2025; Zeng et al., 2025; Chen et al., 2024). While early-stage disease is typically amenable to surgical resection, advanced-stage cases often present limited surgical opportunities, resulting in poorer patient prognosis. Certain cases may also be deemed initially inoperable due to tumor proximity to critical anatomical structures. We present the case of a young female patient with a large high-density mass in the apical segment of the right upper lobe by chest CT, with suspected invasion of the chest wall and other tissues. CT-guided biopsy confirmed lung adenocarcinoma, and molecular profiling revealed an epidermal growth factor receptor (EGFR) mutation. Following neoadjuvant therapy with oral targeted agents combined with chemotherapy, the patient successfully underwent surgical resection. The postoperative course was uncomplicated, with discharge achieved on postoperative day five. This case highlights the potential of targeted neoadjuvant therapy to convert initially inoperable cases to operable status, providing valuable insights for clinical practice.

## Case description

**FIGURE 1:**
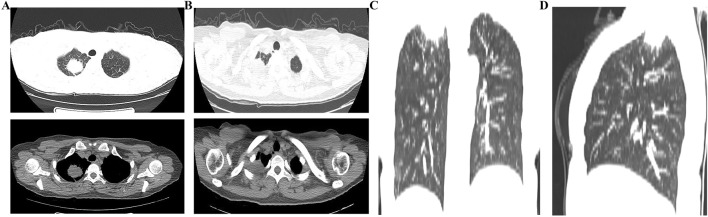
The first plain chest CT scan revealed a space-occupying lesion in the right upper lung. **(A,B)** Lung window and mediastinal window demonstrated a space-occupying lesion in the right upper lobe; **(C,D)** Coronal and sagittal plain chest CT images showed the space-occupying lesion in the right upper lobe.

**FIGURE 2:**
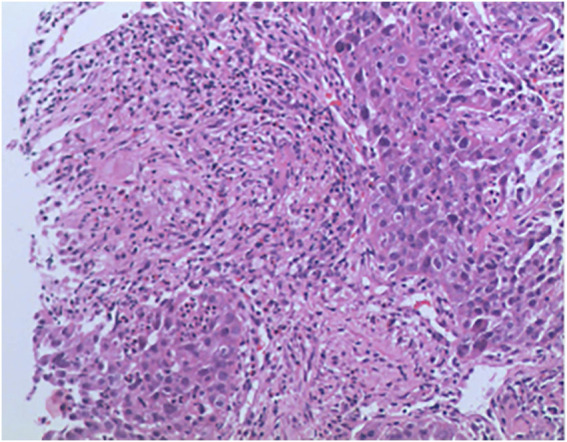
The pathological results after the patient’s puncture biopsy.

The patient is a 33-year-old female. She was examined in our hospital due to cough and expectoration more than 5 months ago. On 13 January 2024, a plain chest CT scan indicated a massive high-density shadow in the apex segment of the upper lobe of the right lung, approximately 4.3 × 3.2 cm in size, which might have invaded the chest wall and other tissues (Figures 1A–D). Direct surgical treatment is difficult, and there is a risk of positive surgical margins during the operation. Therefore, a lung biopsy performed on 18 January 2024, indicated adenocarcinoma of the right upper lung (Figure 2). Genetic testing indicates a mutation at the EGFR site (exon 19 deletion). The patient’s clinical stage is assessed as cT3N0M0. According to the results of the ADAURA trial (Herbst et al., 2023), the patient was treated with oral administration of the targeted drug Osimertinib (80 mg daily). In addition, they underwent chemotherapy (pemetrexed: 800 mg and carboplatin: 500 mg), and the chemotherapy dates were 2024-02-06, 2024-02-28, 2024-03-30 and 2024-04-23 respectively. During this period, the patient reported occasional coughing, expectoration, and mild nausea. No rash, diarrhea, leukopenia, or thrombocytopenia was observed. Antiemetic medication was administered as needed.

**FIGURE 3:**
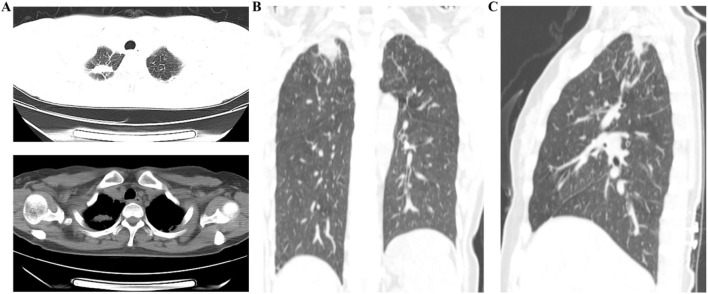
The results of the first plain chest CT scan after treatment. **(A)** Lung window and mediastinal window demonstrated a space-occupying lesion in the right upper lobe; **(B,C)** Coronal and sagittal plain chest CT images showed the space-occupying lesion in the right upper lobe.

**FIGURE 4:**
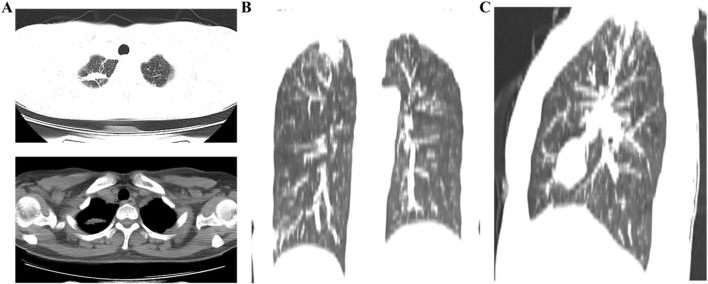
The results of the second plain chest CT scan after treatment. **(A)** Lung window and mediastinal window demonstrated a space-occupying lesion in the right upper lobe; **(B,C)** Coronal and sagittal plain chest CT images showed the space-occupying lesion in the right upper lobe.

On 29 March 2024, a plain chest CT scan of our hospital showed a high-density patch-like shadow in the apex segment of the upper lobe of the right lung, with a diameter of approximately 3.1 × 1.2 cm. It was significantly smaller than before, with blurred edges and was adjacent to the pleura (Figures 3A–C). On 22 April 2024, a plain chest CT scan of our hospital showed a high-density patch-like shadow in the apex segment of the upper lobe of the right lung. The larger area was approximately 3.1 × 1.0 cm, smaller than before, with blurred edges and adjacent to the pleura (Figures 4A–C). Four times after treatment, the patient underwent thoracoscopic right upper lobectomy, thoracoscopic mediastinal lymph node dissection and thoracoscopic pleural adhesion release at our hospital on 13 June 2024.

### Surgical procedure

After general anesthesia, a 3 cm surgical operation hole was made at the 4th intercostal space level along the right midaxillary line. Upon exploration, thoracic adhesions were found. A 2 cm observation hole was made at the 7th intercostal space at the level of the right posterior axillary line to release the adhesions in the right upper lung. No obvious tumor lesions were observed in the right upper lung, but the lung apex was adhered to the top of the pleura, and the boundary with the subclavian artery was unclear. After carefully dissecting the boundary between the apex of the right upper lung and the top of the pleura, the horizontal and oblique fissures of the right lung were opened directly by endoscopy to treat the underdeveloped interlobar fissures. The posterior ascending branch artery of the right upper lung was dissociated along the oblique fissure, the interlobar lymph nodes were dissected, and the posterior ascending branch artery was cut with a linear cutting suture device. The proximal end of the superior pulmonary vein was detached through the anterior mediastinal pleura, and the superior lobe vein was dissected using an endoscopic cutting and suturing device. Below the odd vein, the right upper pulmonary artery trunk was detached, and the tissues around the upper lobe bronchus were fully released. After cutting the anterior superior lobe apex trunk artery with a endoscopic cutting and suturing device, the upper lobe bronchus was treated. The lymph nodes of the 2nd and 4th, hilar, interlobar and others were cleared. Release adhesions in the middle and lower lungs and loosen the ligaments of the lower lungs. The chest cavity was rinsed, sputum was aspirated to expand the lungs, and no obvious air leakage was observed during the water test. One thoracic tube was placed at the 7th intercostal space. After counting the number of pairs, the wound was sutured routinely with fishbone barb thread. The operation was completed. The specimens were sent to the pathological examination after being examined by the family members.

**FIGURE 5:**
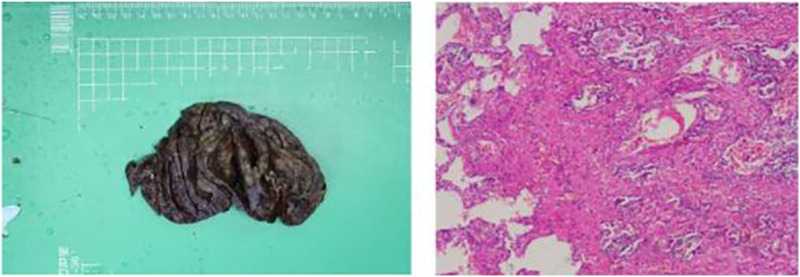
Postoperative pathological specimens and examination results of the patient.

**FIGURE 6:**
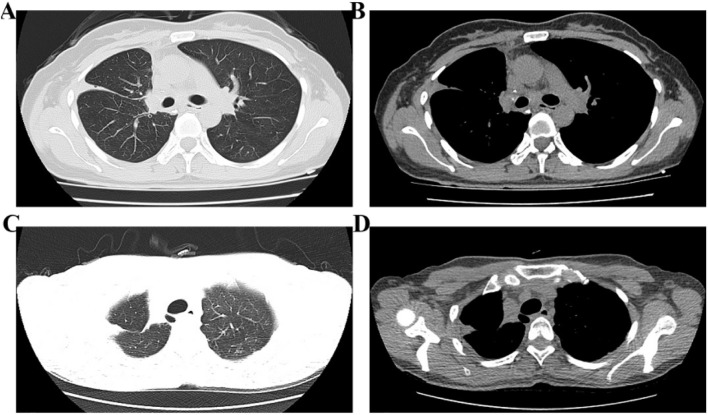
The results of the patient’s chest CT plain scan before discharge and during the follow-up outside the hospital. **(A,B)** Before discharge; **(C,D)** After discharge.

Postoperative pathological examination confirmed a pathological stage of ypT2aN0M0 (Stage IB), with residual invasive adenocarcinoma identified at the tumor bed in the right upper lobe. The surrounding interstitium exhibited fibrous tissue hyperplasia, accompanied by aggregates of foam-like cells, multinucleated giant cells, and cholesterol crystals. No intravascular tumor thrombus or nerve invasion was observed, and no tumor was disseminated along the airway. Cancer involvement was observed in the visceral pleura, while no cancer involvement was found in the bronchial stump and the lung tissue beside the anastomotic nail. No cancer involvement was observed in the superior mediastinal pleura (Figure 5). No lymph node cancer metastasis was observed in the hilar lymph nodes (0/2), and groups 2, 4, 11 and 12 (0/1, 0/1, 0/1, 0/1). On the 4th day after the operation, a plain chest CT scan indicated changes after the resection of adenocarcinoma of the upper lobe of the right lung, with a reduction in right pleural effusion compared to before, and accumulations of inflammation in the posterior part of the lower lobes of both lungs (Figures 6A,B). Symptomatic and supportive treatments such as anti-infection (cefradine), expectoration, antispasmodic, and analgesia were carried out. More than 10 months after the operation, plain chest CT scan in our hospital indicated changes after resection of adenocarcinoma of the upper lobe of the right lung (Figures 6C,D). In addition, according to the patient during our team’s phone follow-up on 1 January 2026, the recent local hospital tests did not indicate any significant disease progression or abnormalities. And the patient has been on adjuvant Osimertinib postoperatively. Tumor marker levels have remained within normal limits, and the ECOG performance status score is 0-1. No significant treatment-related adverse events have been observed during follow-up.

## Discussion

The apical segment of the right upper lung is located at the top of the thoracic cavity, adjacent to important structures such as the subclavian artery, brachial plexus nerve, subclavian vein and the top of the pleura. Its course is closely related to the apex segment. Due to the special growth location of apical adenocarcinoma of the lung, when the lesion is large, it is prone to invade the chest wall or compress adjacent tissues, leading to complex clinical problems. This will increase the difficulty of the operation. The patient was diagnosed with adenocarcinoma of the right upper apical segment of the lung. Given the tumor's close relationship with surrounding tissues and the anticipated difficulty of achieving R0 resection intraoperatively, the patient was not considered a candidate for direct surgery and instead received preoperative neoadjuvant therapy.

After a multidisciplinary consultation, neoadjuvant chemotherapy or targeted therapy can be used to shrink the tumor and reduce the difficulty of surgery. Therefore, we implemented a preoperative neoadjuvant treatment plan, which involves four rounds of chemotherapy combined with osimertinib targeted drug therapy. After active treatment, the patient’s lesion significantly shrank compared to before, and the operation went smoothly. Postoperative re-examination of the chest CT plain scan showed no signs of recurrence.

EGFR mutations are important driver gene variations in lung adenocarcinoma, commonly seen in Asian patients, women and non-smoking patients. Classical mutations (such as exon 19 deletion and L858R point mutation) are sensitive to EGFR tyrosine kinase inhibitors (Tkis) (Roma et al., 2025; Zhang et al., 2023; Liang et al., 2024; Messekher et al., 2025). Currently, first-generation drugs (gefitinib), second-generation drugs (afatinib), and third-generation drugs (Osimertinib) have significantly improved the progression-free survival and quality of life of patients. For example, Messekher et al., reported a case of a patient with EGFR-mutated lung adenocarcinoma diagnosed at stage M1b following surgery. The patient received postoperative treatment with gefitinib and showed no disease progression over a 5-year follow-up period (Messekher et al., 2025). Among them, the third-generation TKI osimertinib has become the preferred first-line treatment due to its ability to penetrate the blood-brain barrier and its inhibitory effect on T790M drug-resistant mutations (Tomizawa et al., 2025; Silva et al., 2025; Ramalingam et al., 2018; Wu et al., 2018). For example, Tomizawa et al., reported that patients with lung adenocarcinoma and brain metastases achieved significantly prolonged overall survival following treatment with EGFR-TKIs (Tomizawa et al., 2025). In this case, it went exactly as we had anticipated. The combination of the third-generation TKI osimertinib and chemotherapy significantly reduced the lesion, providing the patient with a surgical opportunity and promising to prolong the patient’s survival period.

In patients with adenocarcinoma of the right upper apical segment, a large tumor is often closely associated with the surrounding tissues, a feature that significantly affects the treatment strategy. Multidisciplinary collaboration (thoracic surgery, vascular surgery, oncology) and precise imaging assessment are key. Individualized surgery or comprehensive treatment can improve the quality of life and prognosis of patients. Given that this study is a single-case report, its findings should be interpreted with caution and cannot be generalized to a broader patient population. Additionally, In this case, the patient did not undergo contrast-enhanced chest CT or CT angiography (CTA). For such patients, these imaging modalities should be performed to evaluate whether the tumor has invaded adjacent blood vessels. In the future, long-term follow-up of the patient remains essential. This case aims to offer valuable insights and a reference for real-world clinical decision-making.

## Data Availability

The original contributions presented in the study are included in the article/supplementary material, further inquiries can be directed to the corresponding authors.
